# Genome-Wide Studies of *FH* Family Members in Soybean (*Glycine max*) and Their Responses under Abiotic Stresses

**DOI:** 10.3390/plants13020276

**Published:** 2024-01-17

**Authors:** Zhenbiao Zhang, Zhongqi Zhang, Muhammad Shan, Zarmeena Amjad, Jin Xue, Zenglin Zhang, Jie Wang, Yongfeng Guo

**Affiliations:** 1 Tobacco Research Institute, Chinese Academy of Agricultural Sciences, Qingdao 266101, China; 2 Heze Academy of Agricultural Sciences, Heze 274000, China; 3 SINO_PAK Joint Research Laboratory, Institute of Plant Breeding and Biotechnology, MNS University of Agriculture, Multan 60000, Pakistan

**Keywords:** soybean, formins, bioinformatics, abiotic stresses, qRT-PCR

## Abstract

Formins or formin homology 2 (FH2) proteins, evolutionarily conserved multi-domain proteins in eukaryotes, serve as pivotal actin organizers, orchestrating the structure and dynamics of the actin cytoskeleton. However, a comprehensive investigation into the formin family and their plausible involvement in abiotic stress remains undocumented in soybean (*Glycine max*). In the current study, 34 soybean *FH* (*GmFH*)family members were discerned, their genomic distribution spanning the twenty chromosomes in a non-uniform pattern. Evolutionary analysis of the *FH* gene family across plant species delineated five discernible groups (Group I to V) and displayed a closer evolutionary relationship within *Glycine soja*, *Glycine max*, and *Arabidopsis thaliana*. Analysis of the gene structure of *GmFH* unveiled variable sequence lengths and substantial diversity in conserved motifs. Structural prediction in the promoter regions of *GmFH* gene suggested a large set of cis-acting elements associated with hormone signaling, plant growth and development, and stress responses. The investigation of the syntenic relationship revealed a greater convergence of *GmFH* genes with dicots, indicating a close evolutionary affinity. Transcriptome data unveiled distinctive expression patterns of several *GmFH* genes across diverse plant tissues and developmental stages, underscoring a spatiotemporal regulatory framework governing the transcriptional dynamics of *GmFH* gene. Gene expression and qRT–PCR analysis identified many *GmFH* genes with a dynamic pattern in response to abiotic stresses, revealing their potential roles in regulating plant stress adaptation. Additionally, protein interaction analysis highlighted an intricate web of interactions among diverse GmFH proteins. These findings collectively underscore a novel biological function of GmFH proteins in facilitating stress adaptation in soybeans.

## 1. Background

Soybean (*Glycine max* L.) represents one of the major crops in the world, extensively cultivated in the United States, South America, and East Asia [1]. Renowned for its remarkable biological nitrogen-fixing capability achieved through a symbiotic relationship with soil rhizobacteria, soybean has long been acknowledged as a pivotal nitrogen source, supplying essential feed proteins for animals and oils for human consumption [2,3]. Nevertheless, the escalation of severe environmental adversities on a global scale, including water scarcity and soil salinization, has significantly impeded crop growth and agricultural productivity in recent years, exerting a detrimental impact on grain yield and food quality [4,5,6]. To cope with these unfavorable growth conditions caused by drought and salt stress, soybean has evolved complex regulatory mechanisms, particularly during vegetative and reproductive growth stages, enabling its survival and maximizing its achievable yield production. Consequently, extensive efforts in recent decades have been dedicated to elucidating the potential effects of drought and salt stress on various growth stages of soybeans, encompassing impediments to seed germination and seedling growth [7,8,9,10]. Concurrently, the altered expression of numerous genes responsive to drought and salt stress has been observed, attributable to physiological and metabolic alterations. As transcriptome sequencing techniques have rapidly advanced, the acquisition of genome-wide genetic information for soybeans has been realized through the whole-genome shotgun approach. Additionally, the generation of a soybean gene atlas using RNA-Seq has yielded valuable genetic resources, facilitating the exploration of the genetic basis underlying soybean traits [11,12]. Furthermore, bioinformatic analysis based on soybean genomic data has made more contributions to the systematic study of evolutionary relationships and molecular characterization of soybean gene families, offering a potential possibility for the elucidation of molecular biological functions inherent in such genes.

Actin cytoskeleton in eukaryotes plays a pivotal role in cell homeostasis, growth and development, signal perception, and cellular immunity, and its dynamic rearrangement is coordinately regulated by several types of actin-binding proteins (ABPs), functionally categorized into multiple distinct classes, including formins, or formin homology 2 (FH2) proteins [13,14,15,16,17]. Plant formins are relatively evolutionarily conserved in the plant kingdom and generally defined by the conserved FH2 domain. Based on the sequence similarity and domain composition, formins derived from angiosperm are phylogenetically grouped into two distinct clades (Class I and Class II). Members of Class I typically possess two conserved domains: a repetitive, profilin-binding FH1 domain and an actin-nucleating and -capping FH2 domain. On the other hand, Class II members comprise an N-terminal PTEN (Phosphatase and Tensin)-like domain, intricately associated with its phosphatase and fold [18,19,20]. In the model plant *Arabidopsis thaliana* genome, more than 20 formin-encoding proteins have been found, and many of them have been documented the engagement in regulating actin nucleation and dynamics as an important molecular regulator for nucleating apical actin assembly [18,20,21]. Moreover, a polygenetic analysis based on multiple sequence alignment in eukaryotes suggested that formin paralogs exist not only in plant genomes but also in fungi and metazoans. For example, a detailed comparative analysis examined 10 formins in the genome of the *Dictyostelium discoideum* [22]. Beyond the above-implicated formins on fungi and plants, a total of nine distinct formin subtypes were identified in metazoans based on an evolutionary analysis, suggesting a wide existence of the formin family in eukaryotes [23].

Over the past decades, research endeavors have extensively documented the regulatory role of formins in many aspects through control of actin filaments, microfilament, and membrane dynamics, ultimately affecting numerous physiological and cellular processes, especially for cell division, organogenesis, cell-to-cell trafficking, hormone response, and interaction with pathogens. The Arabidopsis plants expressing formin *AFH1* showed an excessive actin cable formation, resulting in tube broadening, growth depolarization, and growth arrest, which emphasizes their crucial role in controlling the normal developmental process of pollen tubes through regulation of actin polymerization [24]. FH3 and FH5, two members of Arabidopsis formins, have been functionally characterized as a regulator of actin dynamics to coordinate actin polymerization and actin filament (F-actin) array construction and finally contribute to proper pollen tube growth [25,26]. Moreover, Arabidopsis FH14, classified as a Class II formin, was reported to impact cell division through its interaction with microtubules and microfilaments. Knock-out mutants of *afh14* showed an abnormal development during the microspore formation process and led to a defective microspore generation, while overexpressing *AFH14* could enhance the resistance of microtubules to oryzalin and cause an imbalance of turnover of spindles and phragmoplasts [27]. In addition, FH13 has recently emerged as a negative regulator of pollen tube growth, as evidenced that mutants lacking *FH13* grow faster than Wild Type (WT) during pollen tube growth, displaying a longer pollen tube, while overexpression of *FH13* leads to a pronounced inhibition of pollen tube elongation [28]. In a parallel context, the actin organizing protein RMD, a type II formin in rice (*Oryza sativa*), was identified as a key component controlling auxin polar transport, auxin distribution gradients, and subcellular localization of auxin transporters through an auxin–RMD regulatory loop to determine the growth and morphogenesis in root cell [15,16,29].

While an increasing body of evidence underscores the significance of plant formins in numerous developmental, physiological processes, and defense responses, there remains a dearth of experimental evidence characterizing their regulatory roles under novel abiotic stress conditions. Moreover, research regarding the plant formin family and its members mainly focuses on Arabidopsis and rice. However, relatively little research on the formins gene family and its members’ functions from other plant species has been conducted so far. Therefore, systematic identification and classification of members of the formins gene family in the soybean need to be conducted further. In the present study, we performed a genome-wide identification of the *GmFH* gene family, encompassing an analysis of physicochemical property, evolutionary relationships, chromosome distribution, gene replication, promoter cis-acting elements, and expression profiles under drought and salt stress conditions, revealing a potential regulatory role of GmFH in response to distinct stress conditions to orchestrate plant growth and development. The findings from our study on the *GmFH* gene family not only contribute to a deeper understanding of their novel biological functions in response to abiotic stress in soybeans but also highlight the functional diversity inherent in plant formins.

## 2. Result

### 2.1. Identification and Characterization of FH Gene Family from Soybean

A comprehensive investigation at the genome-wide level revealed the presence of 34 *GmFH* genes in the soybean (Table 1). The anticipated polypeptides encoded by *GmFH* genes exhibited a substantial variation in length, ranging from 653 to 1464 amino acid residues, with predicted relative molecular weights (RMW) spanning from 72.5 to 162.72 kDa. The isoelectric point (pI) values exhibited a spectrum from 5.42 to 9.43. Notably, all proteins displayed an instability index surpassing 40, indicating their generally unstable nature. The aliphatic index, within the range of 71.48 to 83.73, suggested a notable thermostability among these proteins. The calculated grand average of the hydrophilic index (GRAVY) ranged from −0.378 to −0.61, signifying the hydrophilic nature of the 34 GmFH proteins. Subcellular localization predictions predominantly placed most GmFH proteins within the nucleus, with exceptions observed for GmFH18, GmFH19, GmFH24, GmFH25, GmFH26, and GmFH32, which were predicted to localize in the cytoplasm. These findings collectively contribute to a nuanced understanding of the structural and functional attributes of the identified *GmFH* genes in the soybean.

### 2.2. Evolutionary Comparison of the FH Gene Family from Diverse Plant Species

**Figure 1:**
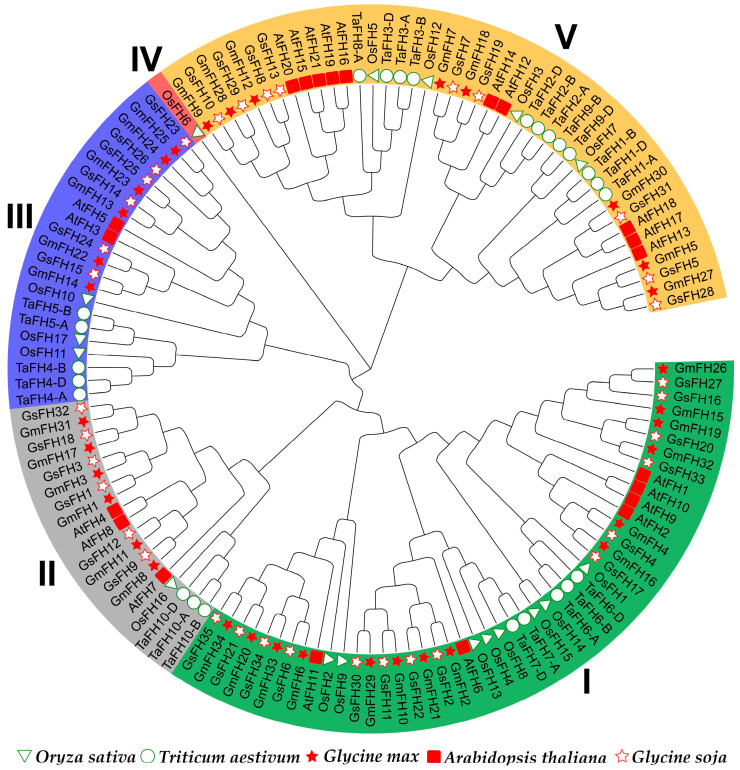
The evolutionary analysis of the *FH* gene family from diverse species groups. The full-length protein sequences were utilized for tree construction. At represents *Arabidopsis thaliana*, Gm represents *Glycine max*, Os represents *Oryza sativa*, Ta represents *Triticum aestivum*, and Gs represents *Glycine soja*. Different groups were presented with different colors.

In an endeavor to deepen our understanding of the evolutionary interconnections within the *GmFH* gene family, FH proteins from diverse plant species, namely *Arabidopsis thaliana*, *Triticum aestivum*, *Oryza sativa*, *Glycine soja*, and *Glycine max*, were incorporated for the construction of an evolutionary tree (Figure 1). The *FH* family members employed for this evolutionary reconstruction are meticulously delineated in Table S1. The outcomes of the evolutionary analysis delineate five discernible groups, denoted as Groups I through V. Groups I and V emerged as the most populous, with Group I encapsulating eleven GmFH members and Group V accommodating eight GmFH, collectively constituting 55.8 percent of the entire GmFH repertoire. In contrast, Group IV exclusively harbored a singular OsFH member. This clustering phenomenon implies homology among FH proteins within the same species, suggesting potential functional similarities.

### 2.3. Analysis of Evolutionary Relationship, Gene Structure, and Conservative Motif of FH Family Members in Soybean

**Figure 2:**
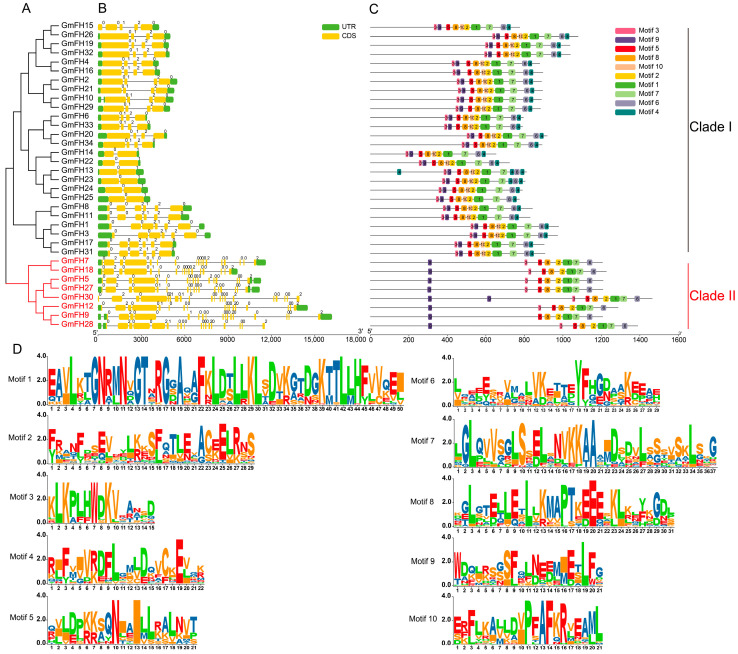
Evolutionary classification, protein domains, and gene structures of *GmFH*. (**A**) Evolutionary analysis of GmFH proteins was performed using MEGA7.0 based on their full-length protein sequences. The resulting evolutionary tree provides insights into the evolutionary relationships among *GmFH* gene family members. (**B**) Gene structures of *GmFH* are depicted, where the scale bar at the bottom indicates the genomic length. Yellow boxes represent exons (CDS), while green boxes denote the 5′ and 3′ untranslated regions (UTRs). Black lines visually represent introns. This representation offers a detailed overview of the genomic organizations of *GmFH* gene. (**C**) The distribution of conserved domains within GmFH proteins is illustrated, emphasizing the various protein lengths and domains. The color-coded scale bar at the bottom facilitates the visualization of distinct domains, contributing to a comprehensive understanding of the structural features of GmFH proteins. (**D**) Amino acid sequences surrounding conserved amino acids are presented as motifs. The letter height in each position reflects the degree of sequence conservation, with taller letters indicating higher relative frequencies of the corresponding amino acids.

To comprehensively scrutinize the structural attributes of *GmFH* gene, an exhaustive examination encompassing conserved domains and exon–intron configurations was conducted on the cohort of 34 *GmFH* genes (Figure 2). The findings elucidate a prevailing presence of conserved domains and untranslated regions (UTRs) across almost all *GmFH* genes, with *GmFH30* constituting the exception (Figure 2B). The intricate composition of GmFH proteins was delineated by identifying various motifs (Figure 2C), where the amino acid sequences surrounding conserved residues manifested as distinct motifs. The overall height of the letters illustrated the graphical representation of sequence conservation at each position proportionate to the relative frequency of the corresponding amino acid. Furthermore, an in-depth analysis of the gene structures of *GmFH* was undertaken (Figure 2D). Results disclosed that *GmFH* genes exhibited variable sequence lengths, with *GmFH9* representing the longest, while *GmFH14* and *GmFH22* featured as the shortest. A predominant inclusion of both 5′ and 3′ untranslated regions (UTRs), exons, and introns was observed in most *GmFH* genes. However, noteworthy exceptions were identified, such as *GmFH13* exclusively possessing 3′-UTRs, *GmFH28* featuring only 5′-UTR, and *GmFH30* lacking any UTRs. Furthermore, the analysis of the conservative motifs of GmFH revealed that all GmFH family members were clustered into two major subgroups (Clade I and II). Most of the GmFH members in Clade I comprised 10 conserved motifs, whereas all members in Clade II contained 8 conserved motifs absent in GmFH30. Collectively, the structural landscape of *GmFH* genes showcased considerable diversity; certain *GmFH* genes within the same clades demonstrated a propensity for comparable exon–intron lengths and numbers. These observations underscore a nuanced evolutionary differentiation within the *GmFH* gene family, concomitant with the retention of relatively similar biological functions among homologous.

### 2.4. Chromosomal Distribution and Duplication Events

**Figure 3:**
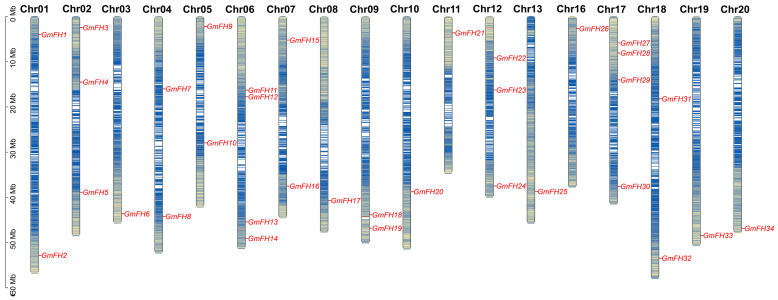
Chromosomal Localization of *GmFH* genes in Soybean. The spatial distribution of *GmFH* genes on soybean chromosomes is illustrated. Each chromosome is labeled at the top with its corresponding chromosome number, providing a clear identification of individual chromosomes. The left-side ruler serves as a scale, representing the length of each chromosome. The gene abundance were indicated by blue colors on chromosome. This depiction offers a visual representation of the specific genomic positions of *GmFH*, facilitating an understanding of the gene’s chromosomal localization within the soybean genome.

The chromosomal disposition and synteny of *GmFH* genes were systematically scrutinized based on their respective genome sequences. The allocation of 34 *GmFH* genes was mapped across the twenty chromosomes of soybeans, revealing a non-uniform distribution pattern (Figure 3). The spatial arrangement of these *GmFH* genes exhibited a dispersed configuration, with varying counts ranging from one to four genes on each chromosome. The spatial arrangement of these *GmFH* genes exhibited a dispersed configuration, with varying counts ranging from one to four genes on each chromosome. Notably, chromosome 06 (Chr06) and chromosome 07 (Chr07) harbored four *GmFH* genes, representing the highest count on all chromosomes, while certain chromosomes harbored only a single member of the *GmFH* family. These findings contribute valuable insights into the chromosomal landscape and synteny patterns characterizing the *GmFH* family, thereby enriching our understanding of their genomic context within soybeans.

**Figure 4:**
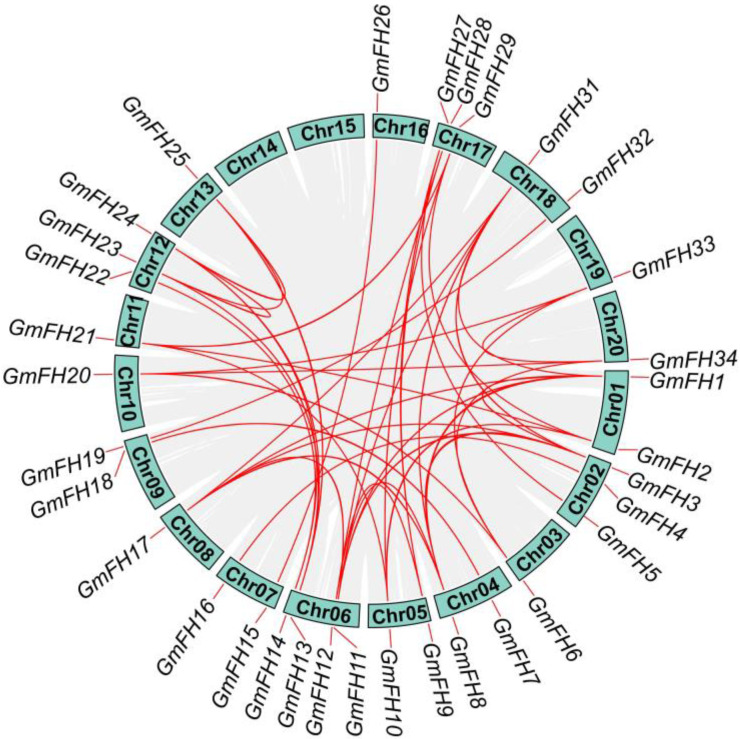
Syntenic relationship among *FH* gene family in soybean. The chromosome numbers are displayed in green boxes. The red lines indicate the syntenic relationship of *GmFH* genes among different gene pairs. The grey lines indicated the syntenic relationship among *GmFH* genes in the soybean genome.

To further enhance our comprehension of the arrangement of genes on each chromosome, an investigation into gene duplication events focused on the *GmFH* family was conducted. Such duplications arise through diverse mechanisms, including tandem and segmental duplication. In this analysis, we discovered 25 segmental duplication pairs existing in the 33 *GmFH* genes but no tandem duplications during this evolutionary investigation (Figure 4). The Ka and Ks ratios were calculated for the identified gene pairs to delve deeper into the evolutionary dynamics (Table 2). Most gene pairs exhibited Ka/Ks ratios less than 1, indicative of robust purifying selection acting upon these *GmFH* genes during their evolutionary trajectory. This pervasive purifying selection implies a concerted effort to preserve the functional integrity of the *GmFH* gene family, underscoring its biological significance and highlighting the evolutionary pressures that have shaped its molecular evolution within the soybean genome.

### 2.5. Investigation of Syntenic Relationships between Soybeans and Other Plant Species

**Figure 5:**
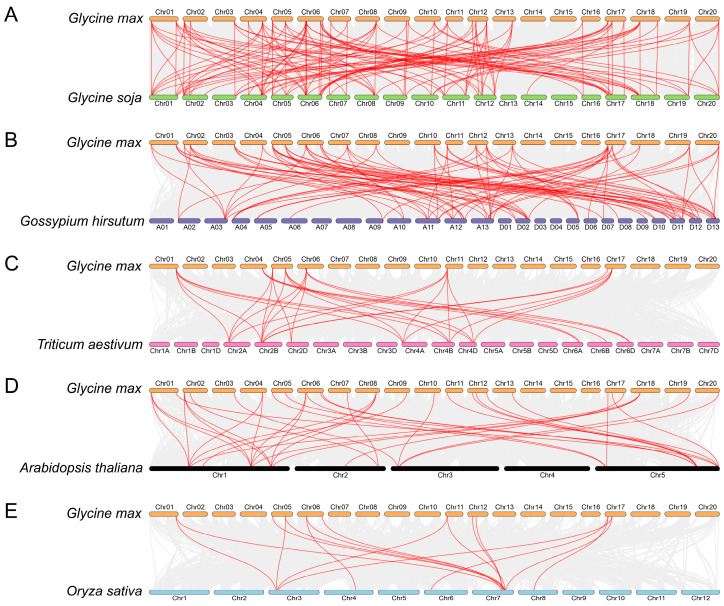
Syntenic relation of the *GmFH* family with the other five plant species, including *Glycine soja* (**A**), *Gossypium hirsutum* (**B**), *Triticum aestivum* (**C**), *Arabidopsis thaliana* (**D**), and *Oryza sativa* (**E**). The highlighted red lines indicate the syntenic gene pairs with *GmFH*. The gray lines indicate the syntenic gene pairs within and other plant genomes. The label next to each chromosome indicates the chromosome number.

The study of syntenic relationships was extended beyond soybean to encompass five distinct plant species, as shown in Figure 5. The outcomes exhibited a total of 32, 25, 9, 27, and 10 *GmFH* genes constituting syntenic gene pairs with *FH* genes in *Glycine soja*, *Gossypium hirsutum*, *Triticum aestivum*, *Arabidopsis thaliana*, and *Oryza sativa*, respectively. Correspondingly, we also found a certain number of collinear gene pairs between these plant species (wild soybean (120), cotton (86), wheat (30), Arabidopsis (37), and rice (16)) and soybean. The comparative analysis unveiled a particularly noteworthy syntenic relationship between *Glycine max* and *Glycine soja*, wherein both species shared an identical set of 20 chromosomes, and their genes exhibited a close syntenic association. This observation not only underscores a high degree of genomic conservation but also implies a close evolutionary relationship, suggesting potential shared physiological functions. Similarly, the syntenic relationship between *Glycine max* and *Gossypium hirsutum* indicated a close evolutionary affinity, with certain genes occupying corresponding chromosomal positions in both plant species (Figure 5B). This alignment further supports the notion of shared ancestry and potential functional similarities.

### 2.6. Analysis of Promoter Cis-Acting Elements in the FH Family Genes

**Figure 6:**
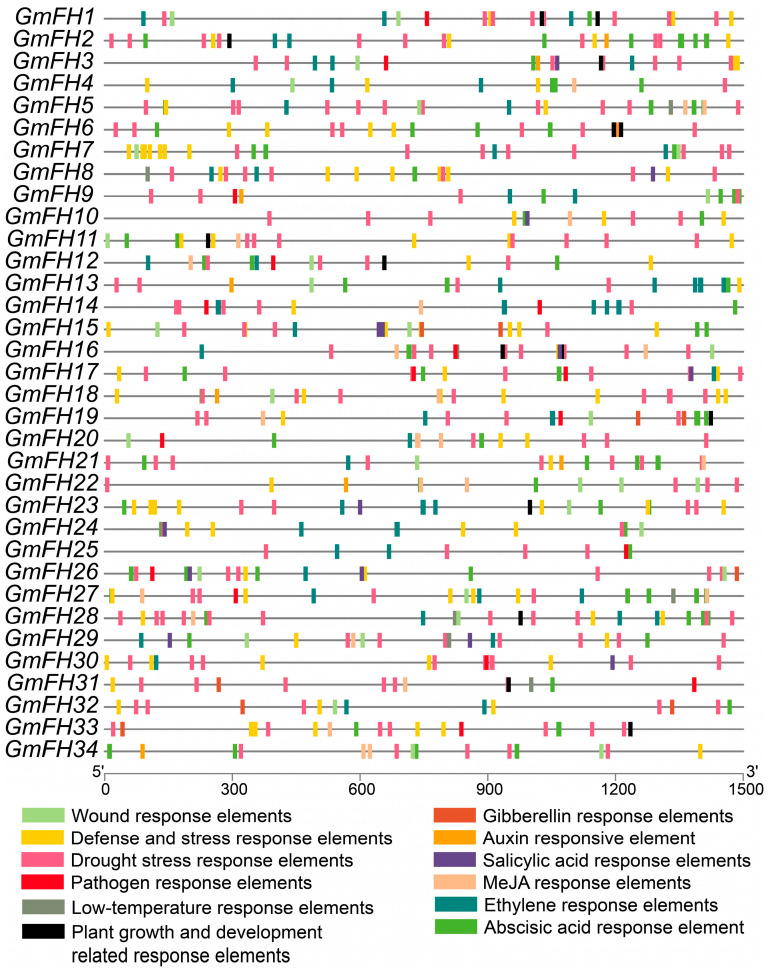
Examination of cis-elements in *GmFH* promoter. The figure presents a comprehensive analysis of cis-elements within the promoters of *GmFH*. The numerical annotations within grids, accompanied by distinct colors, signify the presence of different cis-elements. The spatial arrangement of these elements is specifically focused on those associated with hormonal responses, growth and development, and stress. The variety of colors employed serves to distinguish between different cis-elements, thereby providing a nuanced portrayal of their distribution within the *GmFH* promoter regions.

The scrutiny of cis-elements within gene promoter regions is pivotal for elucidating their biological functions. To unravel the functional underpinnings of GmFH, an in-depth analysis of the cis-elements within these genes was conducted utilizing the PlantCARE databases, and a comprehensive listing is provided in Table S2. These cis-elements were stratified into four overarching categories: hormone-responsive, growth- and development-related, stress-responsive, and wound-responsive elements. Notably, among the hormone-responsive elements, the (MeJA-responsive element) and ABRE (ABA-responsive element) featured prominently, underscoring their significance in the hormonal regulatory milieu (Figure 6).

Similarly, stress-responsive elements, particularly the wound response element, LTR low-temperature-responsive element, and drought stress response element, exhibited substantial representation. The prevalence of these hormone- and stress-responsive elements suggests that GmFH may play integral roles in mediating soybean responses to hormonal cues and environmental stresses. Furthermore, several *GmFH* genes harbor cis-elements associated with pathogen response, such as *GmFH26*, *GmFH20*, and *GmFH31*. This observation suggests a potential involvement of these genes in orchestrating soybean’s response to pathogenic challenges. The meticulous exploration of cis-elements within *GmFH* genes provides a foundation for understanding the regulatory landscape governing their biological functions, with implications for their roles in hormonal signaling, stress responses, and pathogen interactions in the soybean.

### 2.7. Investigation of Networks Depicting Protein-Protein Interactions

**Figure 7:**
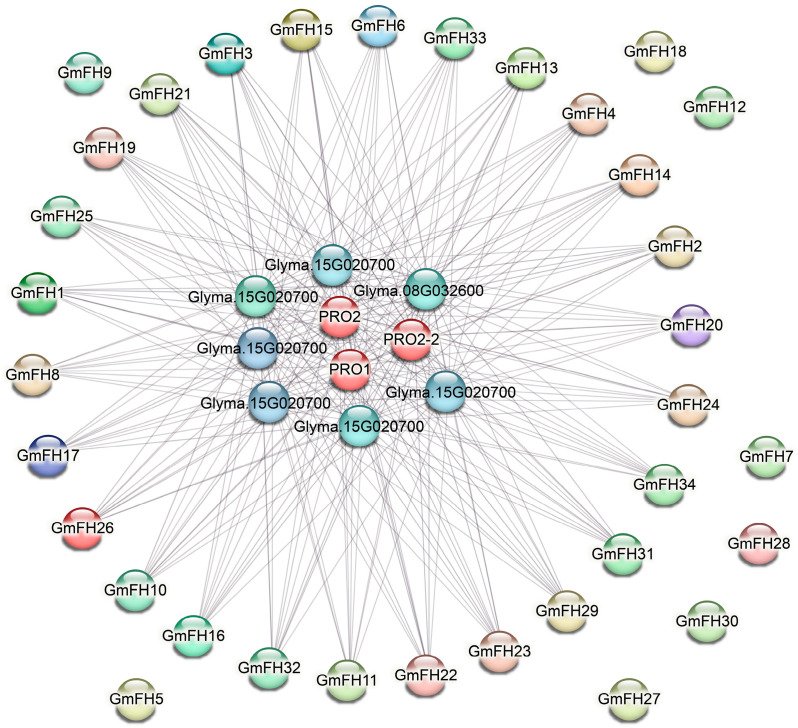
Protein interaction network of the GmFH family. Nodes denote proteins, while edges depict the connections between two proteins. The diversity of edge colors serves to differentiate between types of evidence, thereby indicating distinct forms of functional links. This visual portrayal elucidates the intricate network of protein-protein associations, offering insights into the various functional relationships supported by diverse types of evidence.

The protein-protein interaction (PPI) network analysis of the *GmFH* gene family was executed, and the resultant network is illustrated in Figure 7. Comprising 40 nodes interconnected by edges, this network delineates the intricate web of interactions among diverse GmFH proteins. Notably, the PPI network underscores robust associations among most GmFH proteins, suggesting a cohesive interplay within this gene family. However, a subset of proteins, including GmFH9, GmFH18, GmFH12, GmFH7, GmFH28, GmFH30, and GmFH27, exhibited an absence of interactions. The observed network architecture implies that FH proteins may function as core entities central to the collaborative network of protein interactions. The distinctive clustering of these genes with strong associations hints at the prospect of their concerted involvement in regulating various biological events. Notably, the absence of interactions for specific GmFH proteins may signify unique functional roles or regulatory independence. These findings provide a comprehensive insight into the protein interaction dynamics of the GmFH family and offer a promising avenue for future investigations into the functional roles and regulatory mechanisms of these proteins. The strong associations identified in the PPI network serve as a guidepost for shaping the direction of subsequent functional studies, shedding light on the intricacies of GmFH protein interactions and their implications in the soybean.

### 2.8. Expression Patterns of FH Genes in Soybean Specific to Different Tissues

**Figure 8:**
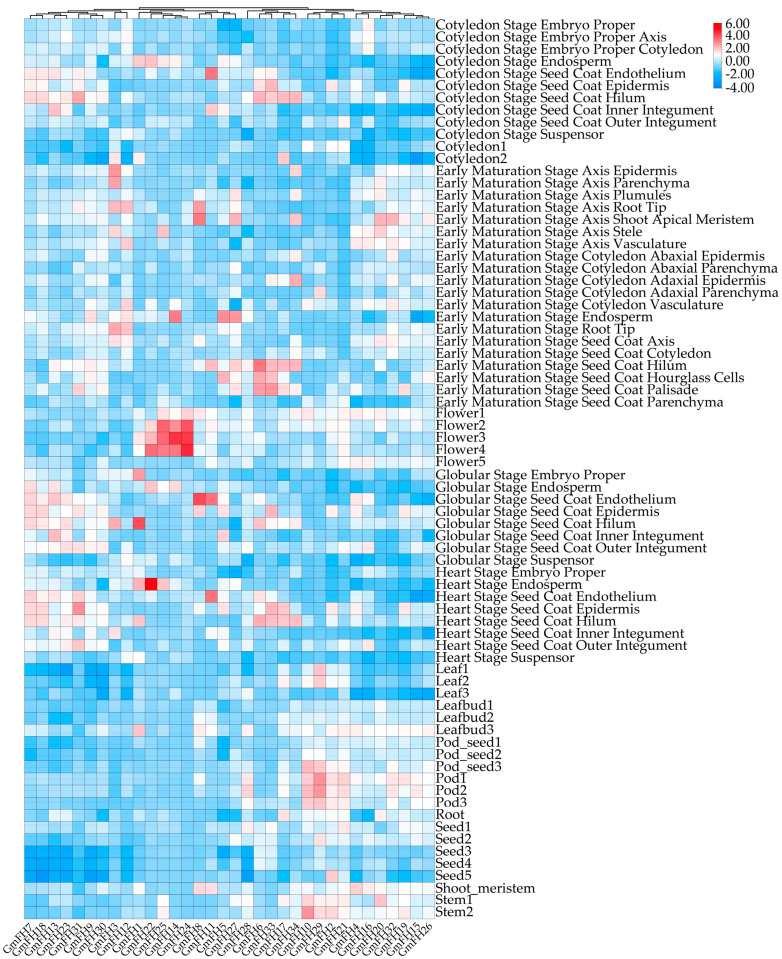
Expression patterns of *GmFH* gene in different plant tissues. Bar indicates different intensities of gene expression in different tissues.

The expression profiles of the *GmFH* family gene were systematically evaluated across diverse plant tissues, revealing distinct and nuanced expression patterns for each gene within the *GmFH* family. This comprehensive analysis encompassed various developmental stages, including the cotyledon stage, early maturation stage, pod stage, root, and shoot. Notably, several *GmFH* family members, including *GmFH24*, *GmFH11*, *GmFH8*, and *GmFH5,* exhibited heightened expression with considerable intensity at the flowering stage. In addition, *GmFH22* demonstrated elevated expression levels in endosperm tissues during the heart stage, while *GmFH1* showcased heightened expression, specifically in the globular stage of the seed coat. Conversely, specific genes exhibited no discernible expression in the diverse tissues under investigation, as indicated by the white coloration in the graph (Figure 8). Specifically, *GmFH7*, *GmFH18*, *GmFH13*, and *GmFH23* manifested suppressed expression levels at the seed stage. The graph’s color-coded bars on the right side visually represented the varying expression intensities of the respective genes across different soybean plant tissues.

### 2.9. Expression Analysis of the FH Family Genes in Soybean in Response to Abiotic Stresses

**Figure 9:**
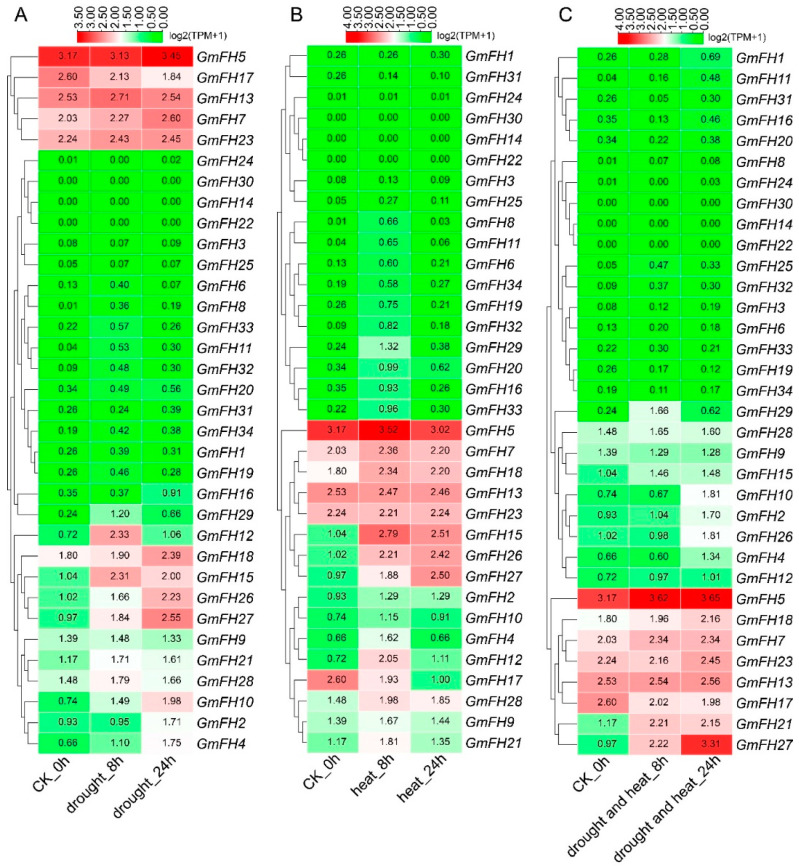
Expression analysis of *GmFH* genes in soybean in response to drought and heat stress. The expression of different genes was examined under drought, heat, cumulative drought, and heat stress. (**A**) Expression profiles of different genes under drought stress after 8 h and 24 h stress treatment. (**B**) Expression profiles of different *GmFH* gene under heat stress treatment of 8 h and 24 h. (**C**) Expression patterns of *GmFH* gene under cumulative heat and drought stress after 8 h and 24 h, respectively.

To evaluate the response of the *FH* gene family in the soybean to abiotic stress, we conducted a comprehensive analysis of the expression levels of each *FH* gene under varying stress conditions. The expression profiles revealed distinct responses of individual *FH* genes to specific stressors. Notably, *GmFH5* exhibited heightened expression levels under drought conditions, with a comparable high expression observed in control plants (Figure 9). *GmFH12* and *GmFH15* displayed relatively elevated expression after 8 h (h) of drought treatment, while *GmFH10* and *GmFH27* exhibited a continuous induction upon drought treatment, indicating a potential role in stress responsiveness. Conversely, *GmFH14*, *GmFH22*, and *GmFH30* demonstrated no detectable expression during both drought treatment and in control plants. In addition, most genes exhibited minimal expression under drought-stress conditions. Under heat stress, *GmFH15* demonstrated significant upregulation after 8 h. Intriguingly, *GmFH17* exhibited elevated expression levels under control conditions but experienced downregulation during heat stress at both 8 h and 24 h. Moreover, *GmFH26* was significantly induced by heat stress in 24 h, but no remarkable difference under both heat and drought stress. The combined heat and drought stress presented distinctive outcomes in the expression profiles. However, *GmFH27* showcased heightened expression under stress conditions, implying a potential role in stress tolerance. Additionally, several *GmFH* genes exhibited consistently high expression levels across control and stress conditions.

**Figure 10:**
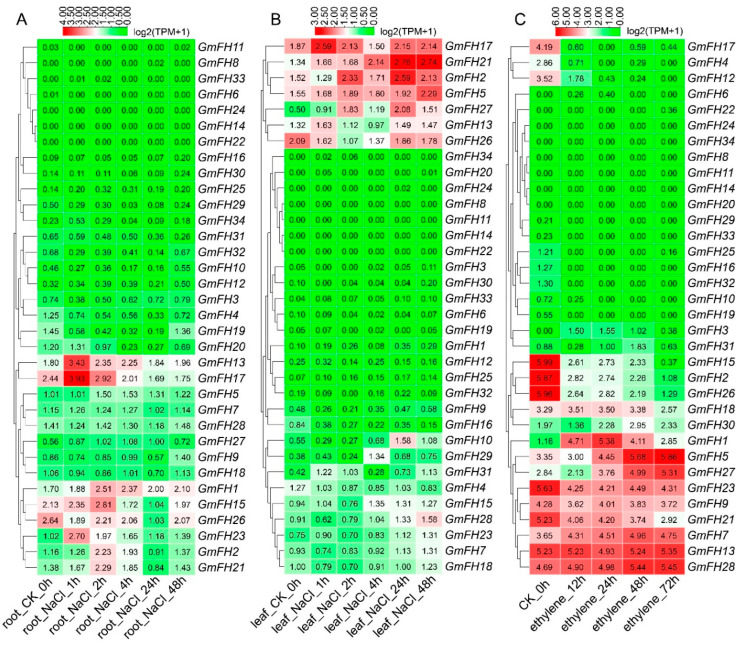
Expression profiles of *GmFH* family gene under salt stress in root and leaf. (**A**) Gene expression patterns of *GmFH* in roots under salt stress treatment after 1 h, 2 h, 4 h, 24 h, and 48 h. (**B**) Gene expression patterns of *GmFH* in leaf under salt stress treatment after 1 h, 2 h, 4 h, 24 h, and 48 h. (**C**) Gene expression profiles of *GmFH* under ethylene treatment after 12 h, 24 h, 48 h, and 72 h.

Examining the *FH* gene family expression dynamics in soybean extended to responses under salt and ethylene stress conditions (Figure 10). We systematically analyzed the expression levels of each *GmFH* gene across diverse tissues subjected to different stress durations, as depicted in Figure 9. Notably, *GmFH13* and *GmFH17* demonstrated heightened expression in roots under salt stress after a 1 h treatment. Conversely, *GmFH17*, *GmFH21* (after 24 h and 48 h treatment), and *GmFH2* exhibited elevated expression levels in leaves under the same stress condition. Upon exposure to ethylene treatment, *GmFH15*, *GmFH2*, and *GmFH26* showcased elevated expression levels in control plants. In contrast, *GmFH23*, *GmFH9*, *GmFH21*, *GmFH7*, *GmFH13*, and *GmFH28* exhibited expression across all ethylene treatments, albeit not very high. Intriguingly, *GmFH5* manifested high expression, specifically in the 48 h and 72 h treatments. Despite the differential responses observed, a predominant trend emerged wherein most genes exhibited low expression levels under salt and ethylene stress conditions. This nuanced exploration of the *GmFH* expression profiles provides insights into their varied responsiveness to distinct abiotic stressors, underscoring the intricate regulatory mechanisms governing stress adaptation in the soybean.

### 2.10. Go Enrichment Analysis of GmFH Family Members

**Figure 11:**
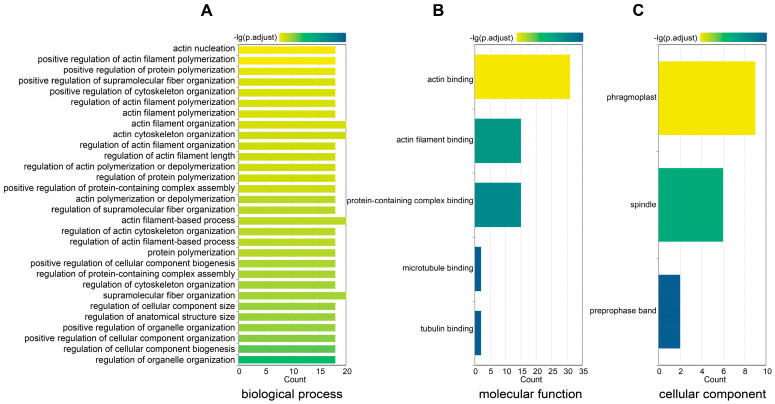
Gene ontology (GO) enrichment for the GmFH gene family. (**A**) GO annotation of GmFH in biological process. (**B**) GO annotation of molecular function. (**C**) GO annotation of a cellular component. The labels on the left represent the GO terms and categories. Different color of boxes indicates different properties.

In-depth functional characterization of the *GmFH* gene family members was pursued to enhance our comprehension of their roles. Functional annotation delineated three distinct categories, each representing different functional dimensions (Figure 11). In the biological process category, the predominant involvement of GmFH was identified in actin filament organization (GO: 0007015), polymerization (GO: 0030838), and nucleation (GO: 0045010). In the molecular function domain, a substantial proportion of GmFH genes demonstrated engagement in protein binding activities, specifically with tubulin (GO: 0015631) and actin filament (GO: 0051015). Within the cellular component category, the prevalent localization of GmFH was observed in the phragmoplast (GO: 0009524), with an additional presence in the spindle (GO: 0005819) and select instances in the preprophase band (GO: 0009574). This systematic functional categorization provides valuable insights into the diverse roles played by *GmFH* gene family members, shedding light on their functional attributes across various biological processes, molecular interactions, and cellular components within the soybean genome.

### 2.11. Verification of Expression Patterns of the GmFH Genes by qRT-PCR

**Figure 12:**
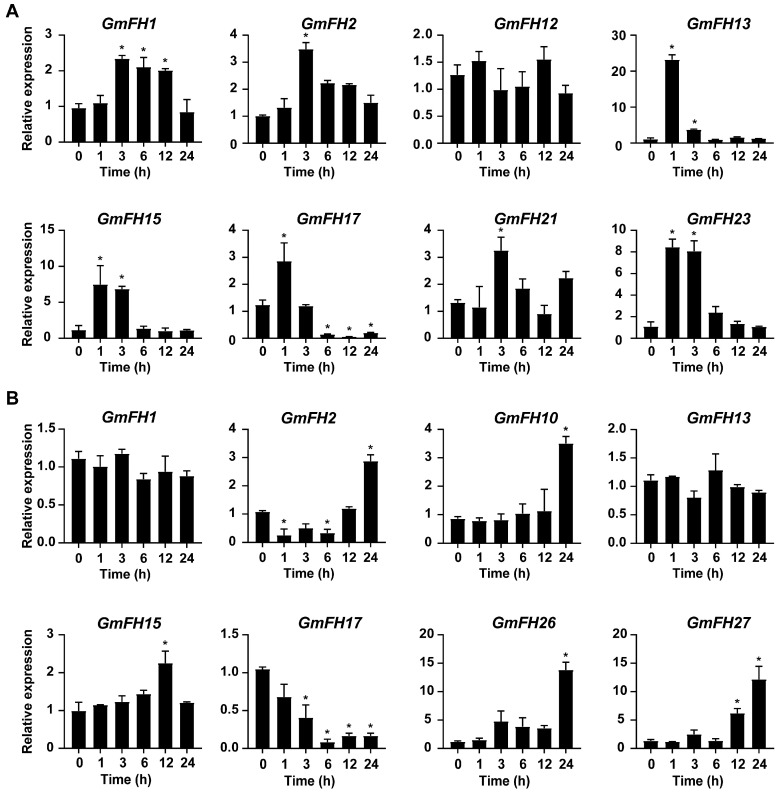
Expression analysis of *GmFH* candidate genes at indicated time points under salt (**A**) and drought (**B**) stress conditions. The analysis was performed with 3 technical replicas for each experiment, and asterisks indicated significant differences, examined by Student’s *t*-test (* 0.01 < *p* < 0.05).

In the present study, several *GmFH* genes exhibiting significant changes in transcriptional levels under salt and drought stress were selected for qRT-PCR analysis, utilizing samples collected at various time points. As a result, the expression patterns of *GmFH* candidate genes examined by qRT-PCR were basically consistent with previous transcriptome data, thereby validating the reliability of existing transcriptome sequencing results. Notably, the majority of *GmFH* genes exhibited a significant increase in expression level at early stages upon salt treatment (Figure 12). Interestingly, *GmFH2* and *GmFH21* were only induced at 3 h after the initiation of salt stress treatment, while the expression of *GmFH17* was dramatically inhibited after 6 h. In contrast, some candidate genes, such as *GmFH10*, *GmFH26*, and *GmFH27*, exhibited up-regulated expression upon drought treatment. In summary, the *GmFH* candidate genes selected for qRT-PCR exhibited diverse expression patterns under salt and drought stress.

## 3. Discussion

The cytoskeleton in eukaryotes primarily consists of two highly conserved polymers known as microfilaments and microtubules. The coordinated reconfiguration and dynamic behaviors of these structures are regulated by various Actin-Binding Proteins (ABPs) [30]. Different ABPs play a distinct but critical role in almost all stages of plant growth and development through the regulation of assembly and disassembly of actin filaments. In plant species, ABPs mainly comprise six kinds, among which formins have been well-characterized actin-bundling proteins defined as a nucleation factor to initiate the microfilament polymerization, afterward participating in the structural organization of the actin cytoskeleton [17]. Previously, formin family proteins have successfully been identified in model plant Arabidopsis and rice, comprising 21 and 16 members, respectively, and an increasing number of formin-encoded genes were recently identified from angiosperm, including tomato, tobacco, rice, pea, sorghum, and wheat. However, a comprehensive study on the identification of putative formin-related genes in the soybean remains missing. Therefore, a genome-wide investigation of formin-encoded genes was conducted by referring to the existing public soybean genome database [18,19,31].

In this analysis, we identified 34 *GmFH* genes distributed non-uniformly across 20 soybean chromosomes (Table 1 and Figure 3), ranging from one to four genes on each chromosome. However, no discernible correlation was observed between the abundance of *GmFH* genes and the respective lengths of the chromosomes, emphasizing a nuanced and intricate genomic organization within the *Glycine max* genome. Formin proteins structurally contain two conserved domains: the profilin-rich FH1 domain, required for the elongation of actin filaments, and the microfilament-nucleating FH2 domain, responsible for actin filament nucleation [25]. Many formin proteins have been functionally documented as nucleation factors guiding transmembrane anchorage of the formins or sometimes localizing formins in endosomes and the tonoplast [32,33,34,35]. Consistent with this, our results to predicate the subcellular localization indicated that all GmFH proteins exhibited either nucleus or chloroplast localization (Table 1), suggesting a putative function as nucleation factors responsible for actin filament nucleation. However, many formin proteins, such as AtFH14, were predominantly localized to the microtubules, strongly suggesting a functional difference between the two kinks of formin proteins.

Formins, characterized by the presence of FH2-containing domain proteins, constitute an evolutionarily conserved family widely distributed in eukaryotes. Evolutionary comparison among five diverse plant species revealed that the *FH* gene family primarily clustered into five main classes according to their evolutionary relationships (Figure 1). FH proteins from discots, including *Arabidopsis thaliana*, *Glycine soja*, and *Glycine max*, demonstrated closer evolutionary conservation, compared with monocots *Triticum aestivum* and *Oryza sativa*, consistent with the previous discoveries, suggesting a relatively conserved mechanism during the evolution of *FH* gene family. A noteworthy clustering pattern was observed wherein FH proteins from *O. sativa*, *A. thaliana*, *G. soja*, *G. max*, and *T. aestivum* were predominantly coalesced within species-specific groups. Specific FH proteins from these five distinct species exhibited clustering, indicative of a closely entwined evolutionary relationship within the FH family. These findings contribute valuable insights into the intricate evolutionary dynamics of the *GmFH* gene family and its evolutionary affinities across diverse plant species. Furthermore, analysis of the syntenic relationship among five species pairs highlighted the closest evolutionary affinity between *Glycine max* and *Gossypium hirsutum* during FH family evolution except for *Glycine soja*, again contributing to the high degree of evolutionary conservation and underscoring the similar biological feathers in homologous gene pairs. Intriguingly, the results also revealed a distinctive pattern wherein soybeans exhibited greater syntenic convergence with dicots compared to monocots, elucidating a nuanced aspect of evolutionary divergence between these plant lineages. Additionally, distribution events suggested that the FH family mainly undergoes segment duplication as a primary driving force contributing to gene expansion. These findings provide valuable insights into the genomic dynamics and evolutionary relationships between soybeans and diverse plant species, thereby enriching our understanding of soybeans’ genetic landscape and evolutionary history.

Analyzing the expression profiles based on previous transcriptome data provides insights into the potential biological functions of GmFH across diverse plant tissues. The examination unveiled dynamic variations in *GmFH* gene expression across different plant tissues and developmental stages (Figure 8). Notably, the distinctive expression patterns of each *GmFH* gene underscored remarkable heterogeneity, with some genes exhibiting robust expression in specific plant parts while displaying no expression in others. For example, expression levels of *GmFH24* and its homolog *GmFH25* were significantly induced at the flowering stage, implying their association with flower organ development. In contrast, *GmFH22* exhibited dramatic increases during the heart stage, especially in endosperm tissues, strongly suggesting a functional involvement during endosperm development. This variability in gene expression within the *GmFH* family across various plant tissues suggests a finely tuned and tissue-specific regulatory framework governing the transcriptional dynamics of these genes in the soybean. To gain an in-depth understanding of the biological functions involved by GmFH in diverse biological processes, a comprehensive analysis focused on their promoter cis-acting elements was performed (Figure 6). The examination identified a large body of hormone-responsive elements, with ABA- and ethylene-responsive elements prominently enriched in the promoter region of the majority of *GmFH* genes. The regulatory roles of ABA in modulating osmotic and drought stress through ABA-dependent or -independent strategies have been well-known in multiple plant species [36]. This result indicates a possible biological process involved by GmFH in responding to ABA-mediated drought stress tolerance. Correspondingly, ethylene is generally regarded as an important signaling molecule modulating salinity tolerance in plants through a fine-tuning of ROS production and removal [37,38]. Salinity can trigger the biosynthesis of ethylene in a plant by regulating the expression of key enzyme genes, and the production of ethylene, in turn, counteracts the salt-induced inhibition in multiple biological processes. The observation of an abundance of ethylene cis-acting elements predicted on the *GmFH* promoter strongly suggested a regulatory role of GmFH in response to salinity stress through a potential ethylene signaling pathway. Furthermore, a certain number of stress-responsive elements specifically respond to drought and low-temperature stresses, such as anaerobic induction element (ARE), stress-responsive element (TC-rich repeats), and drought-inducibility (MYB, MYC, Myb-binding site, MYB-like sequence), were also found on the promoter of some *GmFH* genes, underscoring their biological significance on the enhancement of plant adaptation to adverse conditions.

Formins, being evolutionarily conserved proteins, play crucial roles in nucleation, capping, and bundling of actin filaments, thereby influencing the organization and dynamics of the actin cytoskeleton. Previous research has extensively documented the roles of plant formin proteins in remodeling the actin cytoskeleton to coordinate physiological and cellular changes. In Arabidopsis, AtFH1 has been suggested to play a regulatory role in the normal development of pollen tubes, as overexpression of AFH1 resulted in growth depolarization and growth inhibin [24]. AtFH8 is speculated to regulate the growth of polarized cells, affecting the root hair cell development [39]. In rice, OsFH5, a type II formin nucleating factor, was involved in plant morphology through reorganization and dynamics of the actin cytoskeleton, while OsFH15, encoded by a class I formin, affects grain size by regulating cell expansion [16,40]. In moss *Physcomitrella patens*, For2 (Group II formins) demonstrated the necessity for maintaining polarized plant cell growth since silencing For2 in plants absence caused severe growth retardation [41]. To further probe the potential biological function of formin proteins in the soybean, a comprehensive analysis regarding gene expression of *GmFH* under diverse abiotic stresses was performed based on the previous transcriptome data. Several *GmFH* genes, like *GmFH12* and *GmFH15*, exhibited a relatively higher expression level upon drought or heat treatment for 8 h (Figure 9), while *GmFH26* and *GmFH27* showed a persistent induction after the same treatment for 24 h, suggesting a nuanced regulatory response to heat stress. GmFH17, on the other hand, exhibited an inhibitory effect on its expression level under drought or heat stress conditions, potentially implying a positive regulatory role modulated by GmFH17 in response to drought or heat stress. In contrast with drought or heat treatment, we also identified certain *GmFH* genes displaying heightened expression under salt stress. Among them, *GmFH17* demonstrated an elevated expression level in both root and leaf tissues upon salt treatment, while *GmFH13* and *GmFH23* were significantly induced only in root tissue after salt treatment. Meanwhile, we noticed that many *GmFH* genes, such as *GmFH10* and *GmFH29,* showed the specific induced expression at 24 and 4 h in root under salt stress, strongly suggesting a dynamic spatial and temporal regulation. Considering that ethylene is a stress-related plant hormone that regulates plant salt tolerance responses, it was observed that several *GmFH* genes were specifically induced by ethylene, including *GmFH5* and *GmFH27*. At the same time, the expression level of *GmFH2*, *GmFH15*, and *GmFH26* is higher in control plants than in treated plants when exposed to ethylene (Figure 10), implying their probable functional distinctions in modulating the ethylene signaling pathway. Finally, qRT-PCR results verified the reliability of existing transcriptome data (Figure 1). These findings illuminate the nuanced and context-dependent regulatory dynamics of the *GmFH* gene family in response to abiotic stressors, providing valuable insights into their putative functions in stress adaptation in the soybean.

## 4. Methods and Materials

### 4.1. Plant Growth Conditions and Stress Treatment

Soybean Williams 82 (*Glycine max*) seeds were obtained from the Institute of Crop Science of the Chinese Academy of Agricultural Sciences. Soybean seeds were soaked with 1% sodium hypochlorite solution (*v*/*v*) for 15 min, followed by washing with deionized water to remove residual solution. To promote seed germination, the surface-sterilized seeds were first incubated on the wet filter papers at 25 °C for 3 days. Next, seedlings with consistent growth were selected and cultivated in half-strength modified Hoagland solution supplied with a 16-h light period and an 8-h dark period at 26 °C for growth. For the salt and drought treatment assay, plants growing to the first trifoliate leaf were placed into a newer half-strength modified Hoagland solution, followed by 150 mM NaCl or 20% PEG6000. Samples from different plant tissues were collected at indicated time points (0, 1, 3, 6, 12, 24 h). Three biological replicates for each sample were mixed at least for RNA extraction, and samples were -frozen with liquid nitrogen and then removed into the refrigerator at −80 °C for further use.

### 4.2. Identification and Physicochemical Analysis of the GmFH Family Members

The genome and its corresponding protein sequence of soybean Williams 82 (*Glycine max*) were downloaded from SoyBase (https://www.soybase.org/, (accessed on 14 December 2009)) (Wm82.a2.v1). The hidden Markov models (HMMs) of the FH domain (PF02181) were downloaded from the InterPro database (https://www.ebi.ac.uk/interpro/, (accessed on 6 January 2023)), and candidate genes of *GmFH* family were searched by simple HMM program (TBtools, (version 1.120), E-value < 1 × 10^−5^) [42]. Subsequently, validation of the GmFH protein domains was implemented in NCBI-CDD (https://www.ncbi.nlm.nih.gov/cdd/, (accessed on 4 January 2017)) and SMART (http://smart.embl-heidelberg.de/, (accessed on 8 January 2021)) database. The physical and chemical properties, including relative molecular weight, theoretical isoelectric point, instability index, aliphatic index, and grand average of hydropathicity, of GmFH proteins were analyzed through the ExPASy program (https://www.expasy.org/, (accessed on 1 January 2022)) [43]. Subcellular localization of the GmFH proteins was predicted on Cell-PLoc 2.0 (http://www.csbio.sjtu.edu.cn/bioinf/Cell-PLoc-2/, (accessed on 17 January 2008)) online tools [44].

### 4.3. Evolutionary Analysis between Glycine Max and Other Plants

The protein sequences of wild soybean W05 (*Glycine soja*) were collected from SoyBase (https://www.soybase.org/, (accessed on 14 December 2009)) (Gsoja_W05.v1). The *Arabidopsis thaliana* genome database was extracted from TAIR (https://www.arabidopsis.org/, (accessed on 17 November 2010)). The genomic data and its annotation of wheat (*Triticum aestivum*) and rice (*Oryza sativa*) were obtained from EnsemblPlants (http://plants.ensembl.org/index.html, (accessed on 7 January 2022)). The FH family members from Arabidopsis, wild soybean, wheat, and rice were identified using the same methods described above, and the protein sequences of identified FHs in different plant species were extracted to construct the evolutionary tree on MEGA 7.0 software (Jone-Taylor-Thornton model) based on the multiple sequence alignment results [45]. All parameters are set as the maximum likelihood method, and the bootstrap replicates were 1000 times. Sequence information is presented in Table S3.

### 4.4. Evolutionary Relationships, Conserved Domain, and Gene Structure of GmFH Family Genes

The evolutionary relationship of the *FH* gene family in the soybean was analyzed using the same methods described above, and the corresponding protein sequences of identified *GmFH* genes were extracted to construct the evolutionary tree by MEGA 7.0 as described above [45]. The conserved motifs of GmFH protein were predicted using MEME Suite 5.4.1 (https://meme-suite.org/meme/doc/meme-format.html?man_type=web, (accessed on 1 July 2015)), and TBtools was employed to visualize the conserved boxes of each *GmFH* gene [46]. The gene structures of *GmFH* were constructed with the online website GSDS 2.0 (http://gsds.gao-lab.org/, (accessed on 12 October 2014)), referring to the soybean annotation file [47].

### 4.5. Chromosomal Mapping and Gene Duplication

The chromosome locations of *GmFH* genes were analyzed through a gene location visualization program of TBtools based on the physical positions of the *GmFHs* in the soybean genome [48]. MCScanX program package was utilized to determine gene duplication events of *GmFH* genes, and the collinearity between soybean and other species was analyzed by TBtools (version 1.120) [49]. Next, Ka (non-synonymous rate)/Ks (synonymous rate) values of syntenic pairs of *GmFH* genes were calculated using Kaks Calculator 2.0 software [50].

### 4.6. Promoter Elements, GO Enrichment Analysis, and Protein Interaction

Promoter sequence information (2000 bp upstream of coding sequences (CDS)) of *GmFH* genes was extracted from the soybean genome database, and then its corresponding homeopathic response elements were analyzed by PlantCARE (http://bioinformatics.psb.ugent.be/webtools/plantcare/html/, (accessed on 1 January 2002)) [51]. Subsequently, TBtools were utilized to present the obtained cis-acting regulatory elements [48]. The GO enrichment analysis of the GmFH proteins was conducted using the SoyMD database (https://yanglab.hzau.edu.cn/SoyMD/#/tools/go, (accessed on 9 October 2023)) [52]. The interaction network of GmFH proteins was generated through analysis of GmFH protein information on the online website STRING 11.5 (https://cn.string-db.org/, (accessed on 12 August 2021)).

### 4.7. qRT-PCR Analysis

The RNA extraction of soybean root tissues was performed referring to the manufacturer’s protocol provided from TRIZOL reagent (Invitrogen, Burlington, CA, USA), and cDNA synthesis was performed using PrimeScript™ RT reagent Kit with gDNA Eraser (Perfect Real Time) provided from TaKaRa, Tokyo, Japan. Gene-specific primers used in present studies were designed using primer 5 software and then verified by the NCBI database (https://www.ncbi.nlm.nih.gov/tools/primer-blast/, (accessed on 18 June 2012)). The quantitative expression level was examined on an ABI 7500 real-time PCR detection system referring to the manufacturer’s protocol. Soybean gene *GmActin* (F: CGGTGGTTCTATCTTGGCATC, R: GTCTTTCGCTTCAATAACCCTA) was utilized as the control to normalize the relative expression levels examined using the 2^−ΔΔCT^ method. All primers used for the examination of gene expression were present in Table S4.

## 5. Conclusions

To summarize, 34 *GmFH* genes were identified in this study, and their structural elements, subcellular locations, and evolutionary connections were disclosed. The evolutionary analysis showed a close evolutionary relationship with other species, pointing to possible functional similarities. Segmental duplication became apparent as a plausible mechanism for the gene family’s expansion despite its uneven chromosomal distribution. The *GmFH* promoters showed characteristics linked to different hormones, stress reactions, and developmental processes. The network of protein-protein interactions revealed a robust correlation between their proteins, suggesting a cooperative control of various biological processes. Its importance for additional research is indicated by expression patterns under heat and salt stress, especially the strong response of GmFH1, GmFH15, and GmFH27 to abiotic stress. All things considered, this thorough investigation offers insightful information about the possible functions of *GmFH* gene in biotic and abiotic stress responses, laying the groundwork for further studies to improve crop quality and reduce production losses in challenging circumstances.

## Figures and Tables

**Table 1:** Analysis of the *DUF668* gene family members in soybean. ORF, Open Reading Length. PL, Protein Length. RMW, Relative Molecular Weights. TIP, Theoretical Isoelectric Point. GRAVY, Grand Average of the Hydrophilic Index.

Gene Name	Gene ID	Transcript ID	ORF (bp)	PL (aa)	RMW (kDa)	TIP	Instability Index	Aliphatic Index	GRAVY	Subcellular Localization
*GmFH1*	*Glyma.01G037600*	*Glyma.01G037600.1*	2439	812	88.77	9.32	57.22	82.25	−0.433	Nucleus
*GmFH2*	*Glyma.01G193200*	*Glyma.01G193200.1*	2670	889	97.5	9.22	54.85	72.63	−0.59	Nucleus
*GmFH3*	*Glyma.02G027200*	*Glyma.02G027200.1*	2415	804	88.16	9.43	54.34	82.7	−0.423	Nucleus
*GmFH4*	*Glyma.02G139500*	*Glyma.02G139500.1*	2643	880	97.24	6.36	61.3	74.9	−0.504	Nucleus
*GmFH5*	*Glyma.02G203200*	*Glyma.02G203200.1*	3630	1209	134.08	7.96	58.72	78.02	−0.495	Nucleus
*GmFH6*	*Glyma.03G236400*	*Glyma.03G236400.1*	2388	795	88.79	8.46	45.68	80.06	−0.459	Nucleus
*GmFH7*	*Glyma.04G123600*	*Glyma.04G123600.1*	3621	1206	130.82	8.26	59.26	79.18	−0.407	Nucleus
*GmFH8*	*Glyma.04G177800*	*Glyma.04G177800.1*	1962	653	72.5	7.14	54.4	80.9	−0.477	Nucleus
*GmFH9*	*Glyma.05G024500*	*Glyma.05G024500.1*	3624	1207	135.12	5.42	52.91	78.09	−0.442	Nucleus
*GmFH10*	*Glyma.05G105100*	*Glyma.05G105100.1*	2670	889	98.03	8.98	51.62	76.99	−0.47	Nucleus
*GmFH11*	*Glyma.06G187100*	*Glyma.06G187100.1*	2172	723	79.85	8.37	50.15	83.73	−0.385	Nucleus
*GmFH12*	*Glyma.06G196800*	*Glyma.06G196800.1*	3864	1287	143	6.32	54.37	76.37	−0.477	Nucleus
*GmFH13*	*Glyma.06G266000*	*Glyma.06G266000.1*	2937	978	105.73	7.93	55.26	80.7	−0.378	Nucleus
*GmFH14*	*Glyma.06G301700*	*Glyma.06G301700.1*	2529	842	91.55	6.18	52.57	78.21	−0.516	Nucleus
*GmFH15*	*Glyma.07G058400*	*Glyma.07G058400.1*	2331	776	85.3	5.83	61.38	78.25	−0.546	Nucleus
*GmFH16*	*Glyma.07G206000*	*Glyma.07G206000.1*	2691	896	99.13	6.57	62.96	74.01	−0.537	Nucleus
*GmFH17*	*Glyma.08G292600*	*Glyma.08G292600.1*	2373	790	85.75	9.33	52.68	81.47	−0.454	Nucleus
*GmFH18*	*Glyma.09G215100*	*Glyma.09G215100.1*	3681	1226	132.5	8.69	61.14	77.9	−0.4	Chloroplast
*GmFH19*	*Glyma.09G246200*	*Glyma.09G246200.1*	3114	1037	112.84	6.81	70.19	75.64	−0.41	Chloroplast
*GmFH20*	*Glyma.10G152300*	*Glyma.10G152300.1*	2760	919	101.62	6.73	59.57	80.39	−0.502	Nucleus
*GmFH21*	*Glyma.11G048600*	*Glyma.11G048600.1*	2688	895	98.36	9.33	52.58	72.79	−0.61	Nucleus
*GmFH22*	*Glyma.12G102700*	*Glyma.12G102700.1*	2493	830	90.54	6.57	54.96	77.07	−0.499	Nucleus
*GmFH23*	*Glyma.12G136800*	*Glyma.12G136800.1*	2922	973	105.28	8.78	56.68	79.32	−0.388	Nucleus
*GmFH24*	*Glyma.12G215400*	*Glyma.12G215400.1*	2712	903	99.99	8.55	48.17	79.47	−0.503	Chloroplast
*GmFH25*	*Glyma.13G286100*	*Glyma.13G286100.1*	2724	907	100.17	7.46	49.49	79.89	−0.474	Chloroplast
*GmFH26*	*Glyma.16G027400*	*Glyma.16G027400.1*	3240	1079	117.13	8.61	72.52	73.03	−0.534	Chloroplast
*GmFH27*	*Glyma.17G074500*	*Glyma.17G074500.1*	3627	1208	134.35	8.47	57.2	78.23	−0.52	Nucleus
*GmFH28*	*Glyma.17G102800*	*Glyma.17G102800.1*	4170	1389	152.57	6.39	63.01	71.48	−0.532	Nucleus
*GmFH29*	*Glyma.17G161400*	*Glyma.17G161400.1*	2655	884	97.56	9.21	54.7	73.35	−0.533	Nucleus
*GmFH30*	*Glyma.17G222600*	*Glyma.17G222600.1*	4395	1464	162.72	6.29	52.35	82.6	−0.419	Nucleus
*GmFH31*	*Glyma.18G130800*	*Glyma.18G130800.1*	2325	774	84.56	9.23	53.04	82.78	−0.457	Nucleus
*GmFH32*	*Glyma.18G246800*	*Glyma.18G246800.1*	3120	1039	113.58	6.69	70.01	76.25	−0.413	Chloroplast
*GmFH33*	*Glyma.19G234000*	*Glyma.19G234000.1*	2376	791	88.21	8.24	48.31	80.34	−0.468	Nucleus
*GmFH34*	*Glyma.20G236100*	*Glyma.20G236100.1*	2679	892	98.55	7.23	59.97	78.46	−0.51	Nucleus

**Table 2:** The ratio of Non-synonymous substitution (Ka) and Synonymous substitution (Ks) of *GmFH* segment duplication pairs.

Gene Pairs	Ka/Ks	Gene Pairs	Ka/Ks
*GmFH1/GmFH3*	0.31	*GmFH9/GmFH27*	0.18
*GmFH1/GmFH8*	0.19	*GmFH9/GmFH28*	0.30
*GmFH1/GmFH11*	0.22	*GmFH10/GmFH21*	0.19
*GmFH1/GmFH17*	0.36	*GmFH10/GmFH29*	0.19
*GmFH1/GmFH31*	0.19	*GmFH11/GmFH17*	0.21
*GmFH2/GmFH10*	0.22	*GmFH11/GmFH31*	0.26
*GmFH2/GmFH21*	0.17	*GmFH12/GmFH28*	0.25
*GmFH2/GmFH29*	0.20	*GmFH13/GmFH23*	0.30
*GmFH3/GmFH8*	0.20	*GmFH13/GmFH24*	0.37
*GmFH3/GmFH11*	0.25	*GmFH13/GmFH25*	0.33
*GmFH3/GmFH17*	0.36	*GmFH14/GmFH22*	0.21
*GmFH3/GmFH31*	NaN	*GmFH14/GmFH24*	0.19
*GmFH4/GmFH16*	0.20	*GmFH15/GmFH26*	0.08
*GmFH5/GmFH27*	0.26	*GmFH17/GmFH31*	NaN
*GmFH6/GmFH20*	0.26	*GmFH19/GmFH31*	0.32
*GmFH6/GmFH33*	0.33	*GmFH20/GmFH33*	0.23
*GmFH6/GmFH34*	0.28	*GmFH20/GmFH34*	0.22
*GmFH7/GmFH18*	0.17	*GmFH21/GmFH29*	0.20
*GmFH8/GmFH11*	0.16	*GmFH23/GmFH24*	0.32
*GmFH8/GmFH17*	0.21	*GmFH23/GmFH25*	0.30
*GmFH8/GmFH31*	0.24	*GmFH24/GmFH25*	0.33
*GmFH9/GmFH12*	0.24	*GmFH33/GmFH34*	0.24

## Data Availability

Data are contained within the article and Supplementary Materials.

## Supplementary Materials

The following supporting information can be downloaded at: https://www.mdpi.com/article/10.3390/plants13020276/s1, Table S1: Sequence Information on the *FH* gene family in diverse plants. Table S2: Primer sequence. Table S3: Gene ID and its responding FH gene number in different plants. Table S4: Analysis of *GmFH* primer elements.
